# ID 222 - Colangite Biliar Primária no Brasil: análise de tendências e padrões regionais da mortalidade, 2000 a 2022

**DOI:** 10.5327/2237-9622.2025.v34s1.222

**Published:** 2025-11-25

**Authors:** Letícia Gavioli, Adriane Lopes Medeiros Simone, Bruna Bento dos Santos, Stéfani Sousa Borges, Daniela Oliveira de Melo, Ana Laura de Sene Amâncio Zara

**Keywords:** colangite biliar primária, taxa de mortalidade, estudos ecológicos, estudos de séries temporais

## Abstract

**Introdução:**

A colangite biliar primária (CBP) é uma doença hepatobiliar crônica inflamatória autoimune que, se não tratada, pode evoluir para cirrose e óbito. Dados sobre a doença no Brasil são escassos, dificultando a tomada de decisões baseada em evidências. Não foram encontrados estudos na literatura que estimassem a taxa de mortalidade (TM) da CBP no Brasil. Analisar a TM, bem como suas tendências, pode auxiliar no aprimoramento de políticas públicas sobre a doença. Assim, este estudo teve como objetivo analisar o número de óbitos e a TM por CBP e suas tendências ao longo do tempo.

**Método:**

Foi realizado um estudo ecológico com análise de série temporal dos óbitos por CBP notificados no Brasil, de 2000 a 2022. Foram considerados os códigos da CID-10 que correspondem à CBP: K74.3 (colangite biliar primária), K74.5 (cirrose biliar sem outra especificação) e K83.0 (colangite). As variáveis analisadas foram obtidas a partir dos dados do Sistema de Informações sobre Mortalidade (SIM), por meio do Departamento de Informação e Informática do Sistema Único de Saúde (DataSUS). A taxa de mortalidade por CBP (TMCBP), estimada para cada 100 mil habitantes e padronizada por sexo e idade de acordo com o Censo Brasileiro de 2010, foi estratificada por sexo, faixa etária, causa básica do óbito (CID) e Grandes Regiões. Os dados foram extraídos com o TabWin e analisados no programa IBM Statistics SPSS. Para análise de tendências, visando determinar a taxa incremental média anual (TIMA), foi utilizado o modelo de regressão de Prais-Winsten (p<0,05), utilizando o programa Stata.

**Resultados:**

No período analisado, foram registrados 19.096 óbitos por CBP. A CBP manteve um padrão na ocorrência dos óbitos em toda a série histórica analisada, ocorrendo majoritariamente em pessoas do sexo feminino, acima de 60 anos e com causa básica definida por colangite (K83.0). A TMCBP apresentou tendência crescente (TIMA: 2,36%; IC 95%: 2,04-2,67; p<0,001), variando de 0,30, no ano 2000, a 0,51/100 mil habitantes no ano 2022, sendo superior no sexo masculino, na faixa etária acima dos 60 anos e para o CID K83.0. A região com maior TMCBP foi a Norte (0,74/100 mil habitantes), e a com menor TMCBP foi a Sul (0,48/100 mil habitantes). As maiores TIMA foram encontradas no sexo masculino (TIMA: 3,04%; IC 95%: 2,53-3,55; p<0,001), na faixa etária dos 20 aos 39 anos (TIMA: 2,96%; IC 95%: 1,77-4,17; p<0,001) e nas Regiões Norte (TIMA: 4,03%; IC 95%: 2,26-5,82; p<0,001) e Nordeste (TIMA: 4,55%; IC 95%: 4,04-5,06; p<0,001).

**Conclusão:**

O número de óbitos e a TM por CBP aumentaram no Brasil entre os anos 2000 e 2022. A TM foi elevada na população com 60 anos ou mais, corroborando o padrão de ocorrência da doença nessa população. Apesar de ser uma doença que afeta mais o sexo feminino, a TM no sexo masculino superou a do sexo feminino a partir de 2005, cuja característica é reportada apenas em estudos recentes sobre a doença. A maioria das variáveis apresentou tendência crescente na TM. A diferença nas TIMA para sexo e idade pode ter relação com fatores socioeconômicos, de acesso à saúde e disponibilidade de profissionais médicos capacitados no Brasil.

